# Implementing a Client Reminder Intervention for Colorectal Cancer Screening at a Health Insurance Worksite

**DOI:** 10.5888/pcd11.130276

**Published:** 2014-02-13

**Authors:** Angela M. McFall, June E. Ryan, Polly Hager

**Affiliations:** Author Affiliations: June E. Ryan, Nebrasaka Cancer Coalition, Policy Systems and Environmental Change, Lincoln, Nebraska; Polly Hager, Michigan Department of Community Health, Comprehensive Cancer Control Program, Lansing, Michigan.

## Abstract

**Background:**

Among cancers that affect both men and women, colorectal cancer is one of the leading causes of cancer-related death in Michigan. The American Cancer Society estimates 4,730 new cases and 1,700 deaths due to colorectal cancer in Michigan for 2013. Screening can detect colorectal cancer earlier, when treatment is more successful.

**Community Context:**

The Michigan Department of Community Health represents 1 of 25 states and 4 tribes to receive a multiyear grant from the Centers for Disease Control and Prevention (CDC) to increase colorectal cancer screening rates through population health interventions and clinical services for the underserved. Michigan’s Colorectal Cancer Control Program is implemented in partnership with its Comprehensive Cancer Control Program, which supports the Michigan’s cancer control coalition composed of 114 partner organizations.

**Methods:**

This project had 2 primary objectives: 1) develop a collaborative partnership with 1 Michigan Cancer Consortium organization in which to pilot the intervention and 2) increase colorectal cancer screening rates by implementing a client reminder intervention and measuring the increase in screening rates.

**Outcomes:**

A partnership was established with HealthPlus of Michigan. Of the 95 HealthPlus employees and spouses who received the intervention, 15 completed screening, accounting for a 16% increase in the screening rate. The project was considered successful because both of its objectives were achieved.

**Interpretation:**

Translating evidence-based interventions into practice requires building a relationship with a partner organization, incorporating flexibility, and establishing a realistic timeline.

## Background

Among cancers affecting both men and women, colorectal cancer (CRC) is one of the leading causes of Michigan cancer-related deaths. The American Cancer Society estimates 4,730 new CRC cases and 1,700 CRC deaths in Michigan for 2013. Screening can detect polyps that may become cancerous, so they can be removed. Screening also helps find CRC earlier, when treatment is more successful (1).

To improve health outcomes related to CRC, the Centers for Disease Control and Prevention (CDC) set a goal to increase screening rates to 80% by 2014 (2). The 2012 Behavioral Risk Factor Surveillance Survey estimated that only 69% of eligible Michiganders had an appropriate CRC screening (3). Appropriate screening is defined as a fecal occult blood test within the past year, a sigmoidoscopy within the past 5 years, or a colonoscopy within the past 10 years. As one of the CDC CRC grantees, Michigan is working to increase CRC screening rates by implementing evidence-based public health (EBPH) interventions to meet the national goal.

EBPH, which began to evolve less than 15 years ago, includes making decisions on the basis of available scientific evidence, data, and information systems and on program-planning frameworks; engaging the community in decision-making and sound evaluation; and disseminating what has been learned (4).

EBPH practice integrates science-based interventions with community preferences for improving the community’s health. Before the acceptance of EBPH, many public health interventions were implemented on the basis of leadership preference, political or media pressures, anecdotal stories, or entrenched practices (5,6). Michigan has increasingly incorporated EBPH strategies and practices such as client reminder protocols into its programs and projects (7). Examples of other EPBH strategies incorporated into Michigan’s CRC program are small media, provider assessment and feedback, and one-on-one education.

Client reminders are one EBPH intervention supported by the CDC’s CRC Control Program logic model and by the *Guide to Community Preventive Services* (the *Community Guide*) (www.thecommunityguide.org) to increase CRC screening (8). For these reasons and because the community partners supported their use, client reminders were selected by the project coordinator as an intervention through the Research to Reality Project.

## Community Context

Michigan is a unique state in that it consists of 2 separate peninsulas surrounded by the Great Lakes. Michigan is 1 of the nation’s 10 largest states with more than 9.8 million residents. According to estimates, most Michigan residents are white (76%); the remaining population is African American (14%), Hispanic (5%), Asian (3%), Native American (1%), and unknown/other (1%) (9). A significant Arab American population resides in southeast Michigan (10).

In 2009, Michigan reported 4,802 new CRC cases, representing an age-adjusted rate of 42.9 per 100,000 people. The CRC incidence rate has been declining during the past 20 years; however, a difference remains in the incidence rate between whites and African Americans. When examined by race and sex, African American men have the highest incidence rate, followed by African American women and white men. The lowest incidence rate of CRC is in white women. Comparisons of Michigan CRC data with national composite CRC data from the National Cancer Institute’s Surveillance, Epidemiology, and End Results (SEER) program and with new CDC CRC data indicate that the Michigan CRC rate has been similar to national rates. Each year, approximately 1,800 Michigan men and women die from CRC. In 2010, CRC was responsible for 892 female deaths, a rate of 13.9 deaths per 100,000 women, and 899 men died from CRC, a rate of 18.6 deaths per 100,000 men. The Michigan CRC death rate decreased significantly over the past 20 years as incidence dropped (11).

The Michigan Department of Community Health represents 1 of 25 states and 4 tribes to receive a multiyear grant to increase CRC screening rates through population health interventions and clinical services for the underserved. Funds not directed to population health interventions are used to provide clinical services for low-income uninsured men and women aged 50 to 64 years for CRC screening and follow-up (2).

Michigan implements its CRC program in partnership with the CDC-funded Michigan Comprehensive Cancer Control Program (MCCCP) (12). The MCCCP helps support the state’s cancer control coalition, the Michigan Cancer Consortium (MCC), a statewide organization consisting of 114 partner organizations. The coalition provides a forum for collaboration to reduce the burden of cancer among the citizens of Michigan by addressing cancer prevention and control priorities. In 2010, Michigan received further CDC funding under a new project titled Demonstrating the Capacity of Comprehensive Cancer Control Programs to Implement Policy and Environmental Cancer Control Interventions. This new funding resulted in the development of the MCC Challenge.

The MCC Challenge consists of partner organizations that agreed to assess their health care benefits and employee policies. These organizations have dedicated human resources staff committed to changing policies in their organization, including working with their benefit plan providers to create a healthier and more productive workforce through comprehensive worksite wellness strategies that include increasing cancer screening rates (13). Eight partner organizations participated during the first full year of MCC Challenge implementation in 2012; HealthPlus of Michigan (HP) was 1 of those organizations.

HP is a health and wellness organization that provides customized, nationally recognized health plans. HP manages health care coverage and wellness programs for members residing in Michigan, members of Michigan-based companies who live outside the state, and members of Medicare Advantage and Medicaid. HP formed in 1979 as a nonprofit organization and employs more than 400 employees in full-time or part-time positions in the 3 offices in Michigan. The National Committee for Quality Assurance (NCQA) ranked HealthPlus’s commercial health maintenance organizations (HMOs) among the nation’s top health plans for the past 7 years; its commercial, Medicaid, and Medicare HMO plans are accredited as “Excellent” by the NCQA (14).

The MCC Challenge and the participation of HP provided a unique opportunity for the project coordinator to use this project as the foundation of her involvement with the Research to Reality (R2R) Mentorship Program (https://researchtoreality.cancer.gov/mentorship). The mentorship program provided another level of partnership, an opportunity to gain guidance and perspective from a mentor, and technical assistance from the National Cancer Institute and in the implementation of an evidence-based intervention to address CRC screening. This project had 2 primary objectives: 1) develop a collaborative partnership with 1 MCC organization in which to pilot the intervention and 2) increase CRC screening rates by implementing a client reminder intervention and measuring the increase of screening rates. The purpose of this case study is to describe the progress toward these objectives.

## Methods

To achieve the second objective, it was agreed through ongoing meetings and discussion that HP would use the client reminder system with a population of their choosing (eg, employees, providers, clients). The project coordinator decided to use a single evidenced-based strategy, rather than a combination of strategies shown to increase CRC screening, to simplify the assessment of effect on screening rates. Several project facilitation items were developed including an action plan, a timeline, protocols, and tracking tools. The protocols and tools consisted of the CRC Screening Guideline Protocol (Appendix A), CRC Client Reminder Implementation Protocol (Appendix B), and CRC Evaluation Tool (Appendix C).

The CRC Screening Guideline Protocol defines accepted screening protocols for CRC according to the US Preventive Services Task Force guidelines. Those screening protocols include high-sensitivity fecal occult blood test (FOBT or FIT), flexible sigmoidoscopy, or colonoscopy. People identified with current or past history of CRC or who have had a total colectomy were excluded. The CRC Client Reminder Implementation Protocol provided a timeline with assignments for activities. The CRC Evaluation Tool provided the preintervention and postintervention specifications, definitions, instructions for data analyses and Current Procedural Terminology (CPT) codes. When this project was in the development stage, an MCC partner had verbally committed to the project; however this partner was unable to participate once the project began. The 8 organizations participating in the MCC Challenge during the project timeline were invited to participate in this additional collaboration project on CRC reminders. HP was one organization that indicated interest, and a project description was developed. The project coordinator submitted an application to the Michigan Department of Community Health Institutional Review Board (IRB) for approval. The IRB deemed the project “not human subjects research” with the clarification that “as proposed, this quality improvement project does not involve research or the collection of identifiable information on human subjects.”

The project coordinator held an in-person meeting with HP where several sample client reminder cards were reviewed to determine which may best fit their needs. During discussion of the sample client reminder cards, HP indicated they could not use any of the cards since all mentioned a specific screening procedure, barium enema, that while, medically acceptable as a screening modality, was not acceptable for the insurance company to report for measures of the Healthcare Effectiveness Data and Information Set (HEDIS). HEDIS is a tool used by more than 90% of America’s health plans to measure performance on specific dimensions of care and service. The project CRC Screening Guideline Protocol (Appendix A) incorporated the 2012 HEDIS CRC screening measures, which were essentially the same as those provided in the US Preventive Services Task Force guidelines.

HP’s goal is to increase HEDIS screening rates. Since HP could not count the CRC screening modality, barium enema, for HEDIS, they could not use Michigan’s standard partner materials, which included this procedure. Further discussion determined that HP needed a customized reminder card. In concert with the partners, the Make It Your Own platform (http://miyo.gwb.wustl.edu/about.php) was selected to address this issue. Make it Your Own allows the user to create health information resources tailored to the populations they serve on the basis of tested health communication messages. The organization can modify the message to meet its unique needs. Users of Make It Your Own find culturally appropriate health materials directed at their target population without having to develop them. Using Make It Your Own, HP developed a client reminder postcard tailored to its organizational needs. Once the postcard content was developed, HP obtained the necessary approvals and printed the cards (Figure).

**Figure:**
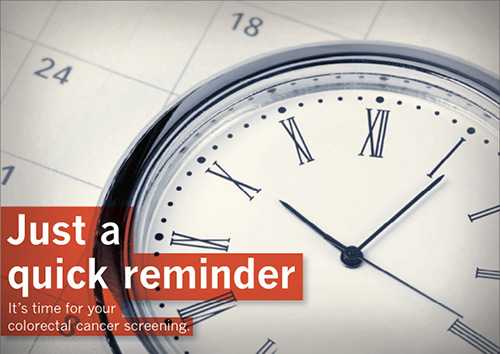

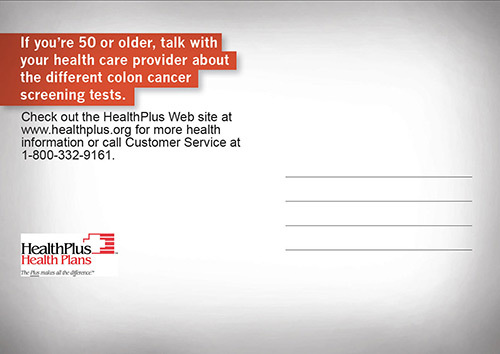
Client reminder intervention for colorectal cancer screening at a health insurance worksite, Michigan Cancer Consortium and HealthPlus of Michigan, Michigan, United States, 2012.

To identify the people who would receive a client reminder postcard, HP staff used CPT codes for CRC screening tests to systematically identify people who did not meet the 2012 HEDIS measures. People without a CPT code indicating an appropriate CRC screening test were identified to receive the intervention. In total, 95 HP employees, eligible spouses, or both were identified as not meeting the 2012 HEDIS measures, and all were sent a one-time client reminder postcard intervention. For Health Information Portability and Accountability Act purposes, HP addressed and mailed the client reminder cards. The MCCCP provided postage to HP for the mailing.

## Outcome

Outcomes for both the partnership and intervention were reached. With regard to the partnership, time delays occurred when the initial partner withdrew and HP agreed to participate in this project. Despite the delays, HP identified value (improved employee health status) in the project and quickly generated a CPT code analysis of employee or eligible spouse CRC screening status. The project coordinator and HP staff developed a working relationship that resulted in project success, including effective problem resolution around an appropriate client reminder card, IRB approval at the Michigan Department of Community Health, and technical assistance from the Make It Your Own staff.

Of the 95 HP employees or eligible spouses sent client reminder cards, 4 (4%) completed screening in the first 3 months and an additional 11 (12%) completed screening after 6 months. The total number of people screened in the first 6 months after the intervention was 15. This number translated into a 16% increase in screening among the target population, which is comparable to a 9% to 20% increase in screening noted by the *Community Guide* with client reminders used for FOBT (15).

## Interpretation

The project was approached with a well thought-out and tested protocol; however, translating evidence-based interventions into practice requires flexibility throughout the process. In this project, the need for flexibility was evident with regard to the timeline and the choice of client reminder cards. Benefits in the choice of collaboration partners were realized, and lessons were learned for replication of similar projects.

Establishing a realistic timeline is an important consideration when implementing an EBPH intervention. The timeline needs to allow for adjustments that may be required as the project unfolds. The project coordinator quickly learned that the timeline that was developed, and necessary to complete this project on the R2R schedule, was not realistic for most of the organizations that were approached. The project coordinator approached HP and they were able to collaborate on the project within the necessary time frame. HP and MCC had an existing relationship that was strengthened through their collaboration on this project and provided opportunities for future projects.

HP had other criteria that were not anticipated, especially regarding the choice of client reminder cards. Although several sample cards were offered, a new reminder card needed to be created to accommodate HP’s specific screening options available for employees that would met HEDIS measures. Once the client reminder card was created, it then needed review internally at HP. Engaging with HP as an insurer added benefit and unique perspective to this project, offering a closed loop between insurer and employer that supported privacy issues. As an insurer, HP had an understanding of HEDIS and routinely uses HEDIS data. These observations support replication of this project by working directly with insurers for client reminders.

Comprehensive cancer control coalition partner relationships could offer a natural platform for replication of this project, whether the desired partner is an insurer or employer. The 68 state, tribal, and territory coalitions are encouraged to consider this EBPH project. Rationale for considering replication includes established relationships with coalition partners, composed of insurers and employers; relative ease considering process and tools used successfully in this project; and low cost, other than staff time. Primary project costs include organization staff time to generate computer reports run of CPT codes (preintervition and postintervention), development and printing of the client reminder cards, and postage to mail the cards.

Overall, the project protocol was validated but could be improved with a few minor changes. Suggested changes include approaching organizations that are members of a statewide cancer control coalition to capitalize on existing partnerships; a longer, more flexible timeline; and earlier submission to the IRB. With these adjustments, we believe similar projects could be conducted to increase CRC screening rates.

## Appendix A. Colorectal Cancer Screening Guideline Protocol

Regular screening, beginning at age 50, is the key to preventing colorectal cancer (16). The US Preventive Services Task Force (USPSTF) recommends screening for colorectal cancer using high-sensitivity fecal occult blood testing, sigmoidoscopy, or colonoscopy beginning at age 50 years and continuing until age 75 years (16).

People at higher risk of developing colorectal cancer should begin screening at a younger age and may need to be tested more frequently. The decision to be screened after age 75 should be made on an individual basis. If you are older than 75, ask your doctor if you should be screened. For more information, read the current colorectal cancer screening guidelines from the USPSTF.

Recommended screening tests and intervals are the following (17):

- High-sensitivity fecal occult blood test (FOBT or FIT), which checks for hidden blood in 3 consecutive stool samples, should be done every year.
- Flexible sigmoidoscopy, where physicians use a flexible, lighted tube (sigmoidoscope) to look at the interior walls of the rectum and part of the colon should be done every 5 years.
- Colonoscopy, where physicians use a flexible, lighted tube (colonoscope) to look at the interior walls of the rectum and the entire colon, should be done every 10 years. During this procedure, samples of tissue may be collected for closer examination or polyps may be removed. Colonoscopies can be used as screening tests or as follow-up diagnostic tools when the results of another screening test are positive. Colonoscopy also is used as a diagnostic test when a person has symptoms, and it can be used as a follow-up test when the results of another colorectal cancer screening test are unclear or abnormal.
- Exclusions, colorectal cancer and total colectomy.

## Appendix B. Colorectal Cancer Client Reminder Implementation Protocol

**Table:**

Activity No.	Activity	Timing
1	The partner agency will generate a list of eligible members by completing coding/data extraction following the criteria to identify eligible members. The criteria to identify eligible members are set forth in the definition of eligible member in the evaluation tool.	April–May 2012
2	With input from MDCH, partner agency will finalize the client intervention (postcard) reminders that will be sent to eligible members.	June 2012
3	MDCH will pay for printing and postage.	June 2012
4	The partner agency will address and mail client intervention to all previously identified eligible members.	June 2012
5	The partner agency will extract data for intervention measurement at baseline and for the same population at 3 and 6 months post intervention. Partner agency will pay for data analysis costs and send measurement results to MDCH. MDCH will share with partners.	October 2012–January 2013
6	The project coordinator will compare data from June (baseline data), October, and January to determine whether partnering agencies rates increased.	June 2012 October 2012 (3-month: July–September 2012) January 2013 (6-month: July–December 2012)

## Appendix C. Colorectal Cancer Evaluation Tool

**Evaluation**

Preintervention: How many eligible members were identified as not completing an eligible screening?

Postintervention: How many eligible members were identified as not completing an eligible screening?

1. At 3 months postintervention?
2. At 6 months postintervention?

**Data analysis**

Preintervention: Baseline data: the percentage and number of eligible members who have not had an eligible screening.

Postintervention:

- Intervention data: the percentage and number of eligible members that had an eligible screening after intervention.
- Baseline, 3-month, and 6-month HEDIS data will be compared to determine an increase in screening rates.

**Definitions**

**Eligible members** are partner agency employees who are aged 50 to 75 years, who have active individual or group medical coverage that is provided or administered by a health plan, and who have not had an eligible screening as defined below. An eligible member excludes individuals with a diagnosis of colorectal cancer or total colostomy within the past 10 years.

The following codes identify excluded individuals:

**Table:**

Description	CPT	HCPCS	ICD-9-CM Diagnosis	ICD-9-CM Procedure
Colorectal cancer	NA	G0213-G0215, G0231	153, 154.0, 154.1, 197.5, V10.05	NA
Total colostomy	44150-44153 44155-44158 44210-44212	NA	NA	45.8

Abbreviations: CPT, Current Procedural Terminology; HCPCS, Healthcare Common Procedure Coding System; ICD-9-CM, International Classification of Diseases, 9th Revision, Clinical Modification; NA, not applicable.

**Eligible screening** as described in the screening guideline protocol is one of the following as identified in the baseline data:

- Fecal occult blood test (FOBT). Regardless of FOBT type, guaiac (gFOBT) or immunochemical (iFOBT) assume that the required number of samples was returned.
- Flexible sigmoidoscopy within the last 5 years.
- Colonoscopy within the last 10 years.

A member/client/patient had an eligible screening if a submitted claim encounter contains any code in the table below:

**Table:**

Description	CPT	HCPCS	ICD-9-CM Diagnosis	ICD-9-CM Procedure	LOINC
FOBT (January 1, 2011–December 31, 2011)	82270, 82274	G0328, G0394	V76.51	NA	2335-8, 12503-9, 12404-7, 14563-1, 14564-9, 14565-6, 27396-1, 27401-9, 27925-7, 27926-5, 29771-3
Flexible Sigmoidoscopy (January 1, 2007–December 31, 2011)	45330-45335, 45337-45342, 45345	G0104	NA	45.24	NA
Colonoscopy (January1, 2002–December 31, 2011)	44388-44394, 44397, 45355, 45378-45387, 45391, 45392	G0105, G0121	NA	45.22, 45.23, 45.25, 45.42, 45.43	NA

Abbreviations: CPT, Current Procedural Terminology; HCPCS, Healthcare Common Procedure Coding System; ICD-9-CM, International Classification of Diseases, 9th Revision, Clinical Modification; LOINC, Logical Observation Identifiers Names and Codes; NA, not applicable.
